# Identification of the Acetylation and Ubiquitin-Modified Proteome during the Progression of Skeletal Muscle Atrophy

**DOI:** 10.1371/journal.pone.0136247

**Published:** 2015-08-24

**Authors:** Daniel J. Ryder, Sarah M. Judge, Adam W. Beharry, Charles L. Farnsworth, Jeffrey C. Silva, Andrew R. Judge

**Affiliations:** 1 Department of Physical Therapy, University of Florida, Gainesville, FL, United States of America; 2 Cell Signaling Technology, Danvers, MA, United States of America; University of Louisville School of Medicine, UNITED STATES

## Abstract

Skeletal muscle atrophy is a consequence of several physiological and pathophysiological conditions including muscle disuse, aging and diseases such as cancer and heart failure. In each of these conditions, the predominant mechanism contributing to the loss of skeletal muscle mass is increased protein turnover. Two important mechanisms which regulate protein stability and degradation are lysine acetylation and ubiquitination, respectively. However our understanding of the skeletal muscle proteins regulated through acetylation and ubiquitination during muscle atrophy is limited. Therefore, the purpose of the current study was to conduct an unbiased assessment of the acetylation and ubiquitin-modified proteome in skeletal muscle during a physiological condition of muscle atrophy. To induce progressive, physiologically relevant, muscle atrophy, rats were cast immobilized for 0, 2, 4 or 6 days and muscles harvested. Acetylated and ubiquitinated peptides were identified via a peptide IP proteomic approach using an anti-acetyl lysine antibody or a ubiquitin remnant motif antibody followed by mass spectrometry. In control skeletal muscle we identified and mapped the acetylation of 1,326 lysine residues to 425 different proteins and the ubiquitination of 4,948 lysine residues to 1,131 different proteins. Of these proteins 43, 47 and 50 proteins were differentially acetylated and 183, 227 and 172 were differentially ubiquitinated following 2, 4 and 6 days of disuse, respectively. Bioinformatics analysis identified contractile proteins as being enriched among proteins decreased in acetylation and increased in ubiquitination, whereas histone proteins were enriched among proteins increased in acetylation and decreased in ubiquitination. These findings provide the first proteome-wide identification of skeletal muscle proteins exhibiting changes in lysine acetylation and ubiquitination during any atrophy condition, and provide a basis for future mechanistic studies into how the acetylation and ubiquitination status of these identified proteins regulates the muscle atrophy phenotype.

## Introduction

Skeletal muscle atrophy and weakness are common responses to many pathophysiological conditions, including muscle disuse [1, 2], cancer [3, 4], AIDS [5, 6] and sepsis [7, 8], yet the molecular and cellular signaling pathways which regulate muscle atrophy and weakness during these conditions are still being defined. However in recent years a critical role of protein acetylation and deacetylation, via lysine acetyltransferase (KAT) and lysine deacetylase (KDAC) enzymes, respectively, has emerged in the regulation of skeletal muscle mass [9–12]. These catalytic enzymes, which add and remove acetyl groups from target proteins, are also more conventionally known as histone acetyltransferases (HATs) and histone deacetylases (HDACs) due to the original discovery that these enzymes regulate lysine acetylation/deacetylation of histone tails to modify chromatin [13]. Yet, in addition to histones, several non-histone proteins, including transcription factors, enzymes, and cytoskeletal proteins are also targets of HATs and HDACs, and acetylation of these target proteins is generally associated with enhanced protein stability [14, 15]. Given the role of increased protein turnover in the regulation of skeletal muscle atrophy, it is perhaps not surprising that several HDAC proteins were recently identified to regulate the muscle atrophy process. Indeed, HDACs 1, 4, 5 and 6 are independently required for skeletal muscle atrophy in response to cast immobilization, denervation and /or chronic angiotensin II signaling [9–12, 16]. However, despite this knowledge, our understanding of the skeletal muscle proteins modified through lysine acetylation/deacetylation during atrophying conditions remains extremely limited.

In addition to reversible acetylation, lysine residues are also subject to ubiquitination. However, in contrast to acetylation which generally increases protein stability, ubiquitination can promote protein turnover. Indeed lysine polyubiquitination is an important step necessary for degradation through the ubiquitin-dependent proteasome pathway (UPP) [17], which is the predominant pathway responsible for the turnover of most cellular proteins during both normal and atrophy conditions [18]. In this regard polyubiquitination of lysine residues on target proteins confers their subsequent recognition for degradation by the 26S proteasome. Interestingly, since lysine acetylation and ubiquitination can have opposing effects on protein stability there is evidentiary support for a regulatory cross talk between these two post translational modifications in the control of protein turnover [14]. In fact, several studies demonstrate a direct inhibitory effect of lysine acetylation on protein degradation through UPP [14, 15, 19]. While direct competition between lysine acetylation and ubiquitination could explain the opposing effects of these modifications on protein turnover, more complex regulatory connections between lysine acetylation and ubiquitination have also been demonstrated [20, 21]. Nonetheless, changes in lysine acetylation and ubiquitination are clearly involved in protein turnover. Thus, a better understanding of the specific proteins and lysine residues modified through these post-translational modifications during conditions of muscle atrophy would offer novel insight into the mechanisms which promote protein catabolism. Therefore, the purpose of the current study was to conduct an unbiased acetylome and ubiquitinome analysis to identify the specific proteins in skeletal muscle which show differential modification via lysine acetylation and ubiquitination during a time course of disuse muscle atrophy.

## Materials and Methods

### Ethics Statement

All animal work was conducted with strict adherence to the guidelines on the recommendations in the Guide for the Care and Use of Laboratory Animals of the National Institute of Health. The protocol was approved by the University of Florida Institutional Animal Care and Use Committee (protocol # 201304285). At the terminal endpoint of experiments, muscles were surgically removed from mice under isofluorane anesthesia and euthanasia subsequently ensured via cervical dislocation.

### Animals

Male Sprague Dawley rats were purchased from Charles River Laboratories (Wilmington, MA) and housed at the University of Florida in a temperature and humidity-controlled facility with a 12-hour light/dark cycle. Water and standard diet were provided ad libitum and access to these was in no way restricted during the immobilization period.

### Hind Limb Cast Immobilization and Tissue Isolation

To induce muscle disuse, the hind limbs of rats were bilaterally cast immobilized with the ankle in the plantar flexed position, as previously described [22–24]. After 2, 4 or 6 days of immobilization, or weight bearing activity for controls, the soleus and plantaris muscles were removed, weighed, and rapidly frozen in liquid nitrogen before storage at −80°C until further biochemical analyses.

### RNA Isolation and Gene Expression Analysis

Total RNA was isolated from the plantaris muscles using the Trizol-based method, as previously described [25]. cDNA was subsequently generated from 1 μg of RNA using the Ambion RETROscript first strand synthesis kit (Life Technologies, Grand Island, NY) and used as a template for quantitative RT-PCR using a 7300 real-time PCR system (Applied Biosystems, Foster City, CA). mRNA expression was assessed using TaqMan probe-based chemistry and the following primers from Applied Biosystems: atrogin-1, GenBank NM_133521; MuRF1, GenBank NM_080903; and 18S, GeneBank X03205.1). Quantification of expression was performed using the relative standard curve method.

### Skeletal Muscle Processing and Peptide Preparation

**Fig 1:**
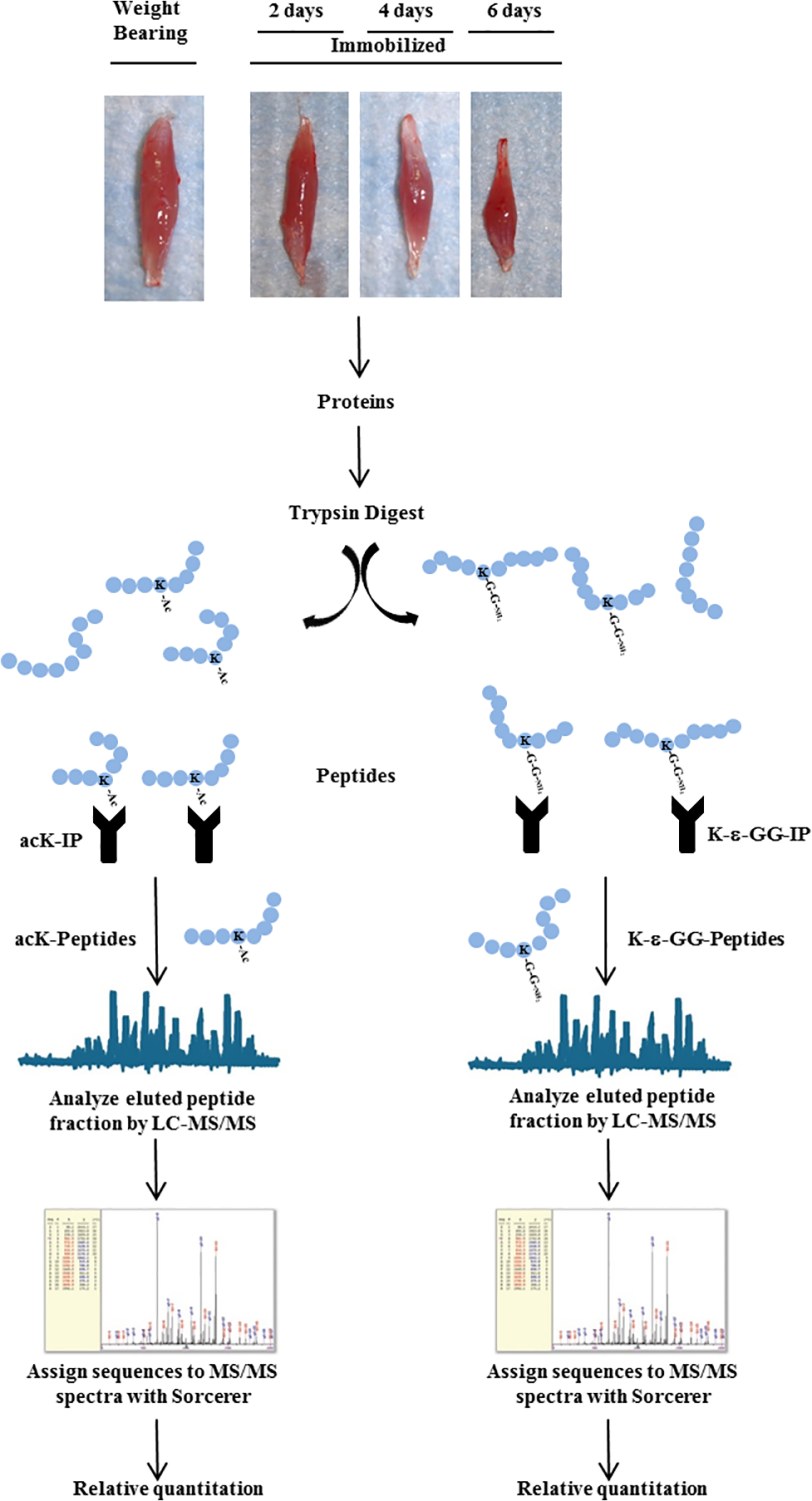
Schematic of procedures for identification and relative quantitation of the acetylome and ubiquitin-modified proteome. Soleus muscles were harvested from weight bearing control, and 2-day, 4-day and 6-day cast immobilized rats. Muscles were homogenized, proteins digested with trypsin, and the resulting peptide fraction desalted. From this peptide fraction acetylated and ubiquitin modified peptides were immunoprecipitated with an anti-acetyl-lysine (K-Ac) motif antibody and a ubiquitin remnant (K-ε-GG) motif antibody, respectively. Immunoprecipitated peptides were separated and analyzed in duplicate by LC-MS/MS using LTQ-Orbitrap-Elite. MS/MS spectra were evaluated using SEQUEST 3G and the SORCERER 2 platform from Sage-N Research. Searches were performed against the NCBI Rattus norvegicus database with mass accuracy of +/-50ppm for precursor ions and 1 Da for product ions. Results were filtered with mass accuracy of +/-5 ppm on precursoe ions and presence of the intended motif.

To obtain sufficient amounts of protein, at least 4 soleus muscles were pooled from separate animals into each of the following groups: weight bearing (control), 2 days immobilized, 4 days immobilized and 6 days immobilized. Each group of pooled soleus muscles was shipped, on dry ice, to Cell Signaling Technology, Inc. (CST Inc, Danvers, MA) for AcetylScan and Ubiscan analysis (**Fig 1**). The tissue samples were processed according to the standard procedures outlined by Cell Signaling Technology with the following specific details. Muscles were homogenized in urea lysis buffer at a ratio of 0.1mg tissue:1ml buffer (9 M sequanol grade urea, 20 mM HEPES, pH 8.0, 1 mM β-glycerophosphate, 1 mM sodium vanadate, 2.5 mM sodium pyrophosphate) using a Polytron tissue homogenizer. Tissue homogenates were then sonicated three times for 20 seconds each at 15 watts output power with a 60 Sonic Dismembrator with 1 minute on ice to cool between each burst. Sonicated lysates were centrifuged 15 min at 12°C at 20,442 × g. Supernatants were collected and reduced with 4.5 mM DTT for 30 min at 55°C. Reduced lysates were alkylated with 100 mM iodoacetimide for 15 minutes at room temperature in the dark. The samples were diluted 1:4 with 20 mM HEPES, pH 8.0, and an equal amount of total protein from each condition was digested to completion via overnight incubation with 10 μg/ml trypsin, treated with tosyl phenylalanyl chloromethyl ketone (Worthington Biochemical Corporation, Freehold, NJ) in 1 mM HCl. Digested peptide lysates were acidified with trifluoroacetic acid (TFA) to 1% final, and peptides were desalted over 360 mg Sep-Pak Classic C18 columns (Waters, Cat # WAT-051910). Peptides were eluted with 40% acetonitrile in 0.1% TFA, lyophilized, and stored at −80°C.

### Enrichment of Peptides Altered by Lysine Acetylation and Ubiquitin-like Modifications

The acetyl-lysine motif antibody (Cat. # 9895; CST, Inc.) and ubiquitin remnant (K-ε-GG) motif antibody (Cat. #8925; CST, Inc) were coupled to protein A agarose beads (Cat. # 11134515001; Roche Diagnostics Corporation, Indianapolis, IN). For each immunoaffinity purification (IAP), 40 μl of packed beads and 1.6 nmol of antibody were used. Antibody was conjugated with beads and incubated overnight at 4°C in PBS (pH 7.4) on a rotator. The beads were then washed with 14 ml PBS (~ 200x the agarose bed volume) four times to remove unbound antibody and then washed with 14 ml of IAP buffer 2X, which consisted of: 50mM morpholinepropanesulfonic acid pH 7.2, 10 mM sodium phosphate dibasic, 50 mM sodium chloride. The antibody/bead slurry was brought to a 1:1 volume and 80 μl of slurry was aliquoted to a 1.7 ml microfuge tube per IAP.

To normalize samples for the subsequent quantitative analysis, the total amount of protein from each sample condition was controlled by processing EQUAL milligram equivalents of each lyophilized tryptic peptide preparation. Equal amounts of total peptide were suspended in 1.4 ml of IAP buffer, placed in a bath sonicator (Branson 2510) for 5 minutes, and then the solubilized peptides were spun at 13,362 x g at 4°C for 10 minutes. The soluble peptides were transferred to 60 μl antibody/bead slurry in a 1.7 ml microfuge tube and then placed on a rotator for 2 hours at 4°C. The antibody/bead mixture was pelleted for 30 sec at 2,000 x g and the supernatant removed and the beads were washed with 1.4 ml IAP buffer three times and then washed twice with 1.4 ml HPLC grade deionized H_2_O. The peptides were eluted from the antibodies with 50 μl 0.15% TFA twice with a 5 minute incubation at room temperature each time. The eluted peptides were pooled, and desalted over p10 tips packed with C18 Empore disks (Cat # 2215; Sigma-Aldrich, St Louis, MO) using the stage tip method described by Rappsilber et. al. [26], and dried under a vacuum.

### LC-MS/MS

Immunoprecipitated peptides were processed and evaluated by Cell Signaling Technologies, Inc. via mass spectrometry as previously described [27–29]. Briefly, immunoprecipitated peptides were resuspended in 0.125% formic acid and separated on a reversed phase C18 column (75-μm inner diameter x10 cm) packed into a PicoTip emitter (~8μm inner diameter) with Magic C18 AQ (100 Åx5μ m). Peptides were eluted using a 96-min linear gradient of acetonitrile in 0.125% formic acid delivered at 280 nl/min. Tandem mass spectra were collected in a data-dependent manner with an LTQ-Orbitrap Velos mass spectrometer running XCalibur 2.0.7 SP1 using a top twenty MS/MS method, a dynamic repeat count of one, and a repeat duration of 35s. Real time recalibration of mass error was performed using lock mass [30] with a singly charged polysiloxane ion m/z 371.101237 [27]. For each sample condition, duplicate injections were obtained. Peptide identity was determined via searches against the NCBI *Rattus norvegicus* database, release date 9/6/2010; containing 29,374 sequences), using SEQUEST 3G and the SORCERER 2 platform from Sage-N Research (v4.0, Milpitas, CA). A precursor mass tolerance of 50 ppm and a product ion tolerance of 1.0 Da (Collision-induced dissociation spectra) or 0.02 Da (higher-energy collisional dissociation spectra) were allowed, as recently used [31]. Mass accuracy of +/- 50 ppm was used for precursor ions and +/- 5 ppm for product ions. Reverse decoy databases were included for all searches to estimate false positive rates and filtered using a 5% false discovery rate in the Peptide Prophet module of SORCERER 2. This was justified by an acceptable ratio of false-positive hits for a discovery project. Search parameters were run with the allowance for the acetylation of lysine residues (variable modifications), oxidation of methionine residues (variable modification) and carboxamidomethylation of cysteine residues (fixed modification). Label-free quantification was performed using proprietary software as previously described [27, 32, 33].

Within **S2 and S6 Tables**, columns labeled “MS2 Spectrum Number” provide numerical spectral values which are hyperlinked to the corresponding spectral images (with corresponding “y” and “b” ion series), which also illustrate the sequence coverage for each of the peptides identified.

### Data Analyses

All quantitative results from the LC-MS/MS were procured by Cell Signaling Technology, Inc. using proprietary software and produced by quantifying the apex peak height or peak area of the corresponding peptide assignments, a method which was previously published [32, 33]. To be clear, this study does not utilize peptide or protein spectral counting, but instead measures peptide signal intensity (peak height or area).

The method of tabulating and filtering acetylation and ubiquitin-like changes between groups was conducted as previously reported [27]. Briefly, quantification was based on a single peptide ion for each identified site of acetylation or ubiquitination. Extracted ion chromatograms for peptides that changed in abundance between samples were manually reviewed to ensure accurate quantitation. Where necessary, fold changes were normalized using a technique in which the median log2 ratio is set to 0 and all fold changes are adjusted relative to the median. The median offset values that were calculated from the log2 ratios for each of the binary comparisons were 0.002, 0.007 and -0.277 (sample B versus control, sample C versus control and sample D versus control, respectively). To identify differentially acetylated or ubiquitinated peptides in muscles from 2, 4 and 6 days of disuse compared to control, the following criteria were used: a coefficient of variance ≤ 50%; a fold change ≥ 2.5 with a signal intensity height ≥ 200,000 or signal intensity area ≥ 500,000, *or* a fold change ≥ 2.0 with a maximum signal intensity (peak height or area) ≥ 1,000,000. Cells with “N.D.” indicate that a fold change for the corresponding peptide was not determined because peptide signal intensity could not be detected.

### Gene Ontology Analysis

Proteins that were increased or decreased in acetylation or ubiquitination at each time point were uploaded into DAVID Bioinformatics database (Maryland, USA) [34, 35] for functional enrichment analysis. To reduce redundancy in annotations we used the functional annotation clustering tool, which groups similar annotations together. The threshold of EASE score, which is a modified Fisher Exact P-value, was set to 0.05 and a minimum count of 2.0.

### Myofibrillar gels, Western blotting and immunoprecipitation assays

Muscles were homogenized in a high-salt lysis buffer to extract and solubilize myofibrillar proteins, as previously described [36, 37]. The buffer consisted of 300 mM NaCl, 0.1 M NaH_3_PO_4_, 0.05 M Na_2_HPO_4_, 0.01 M Na_4_P_2_O_7_, 1 mM MgCl_2_, 10 mM EDTA, and 1 mM DTT (pH 6.5) and complete protease inhibitor cocktail (Sigma-Aldrich). Tissue lysates were centrifuged at 16,000 *g* for 3 min at 4°C, and the supernatant was collected. Protein concentration was determined using the RC-DC Assay (Bio-Rad, Hercules, CA, USA). To determine MyHC abundance, samples were loaded into a 10% polyacrylamide gel (Criterion precast gels; Bio-Rad), which subsequently ran at 200 V for 50 min at 4°C. The gel was stained with Coomassie blue for 3 h (Thermo Fisher Scientific, Waltham, MA, USA) and washed with dH_2_O 3 times. The band corresponding to MyHC was visualized and imaged using an Odyssey Infrared Imaging system (LI-COR, Lincoln, NE, USA). For measurement of actin and myosin light chain 1/3f

Western blot analysis was performed according to standard procedures that have been described previously [23]. Primary antibodies against sarcomeric actin (JLA20; Developmental Studies Hybridoma Bank (DSHB)) and myosin light chain 1 and 3f (F310; DSHB) were used according to the manufacturer's directions. For myosin acetylation assays protein was incubated overnight with either an antibody against sarcomeric myosin (MF20; DSHB), or a non-specific IgG control antibody (no. 2729, Cell Signaling Technology), using the Catch and Release v2.0 Reversible Immunoprecipitation System (no. 17–500, Millipore) as used and described previously [38]. The following day, immunoprecipitated proteins were washed, eluted in denaturing buffer and heated before analysis by western blot. For myosin ubiquitination assays protein was incubated with Agarose-Tandem ubiquitin binding entity (TUBE)2 beads (Life Sensors, Inc.; Cat. # UM402) or control agarose beads (Life Sensors, Inc.; Cat. # UM400), according to manufacturer’s instructions. Immunoprecipitated proteins were washed, spun at 500 *g* for 5 min at 4°C, and eluted in denaturing buffer and heated. Samples were loaded into a 10% polyacrylamide gel, as outlined above, to visualize and image the band corresponding to MyHC.

### Statistical Analysis

Muscle weight and mRNA data were analyzed using a one-way ANOVA followed by Bonferroni post-hoc comparisons where appropriate (GraphPad Software, San Diego, CA). These data are expressed as means ± SE, with significance set at *P* < 0.05.

## Results

**Fig 2:**
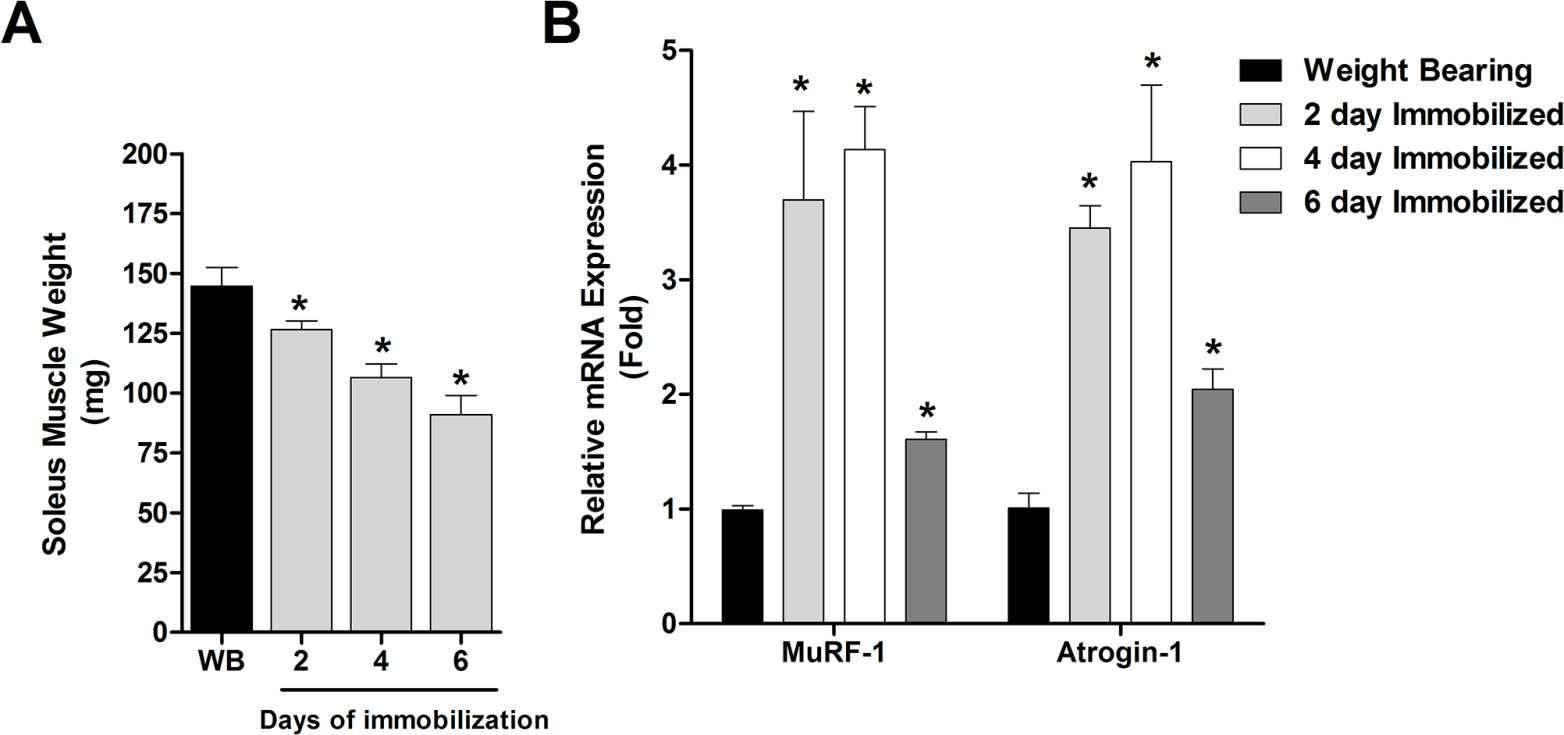
Activation of the muscle atrophy program. Hind limb muscles were harvested following 2, 4 and 6 days of cast immobilization and A) Soleus muscles were weighed to verify progressive muscle atrophy, and (B) Atrogin-1 and MuRF1 mRNA levels were measured in the plantaris muscles. All data are reported as means ± SE, normalized to the control group; n = at least 5 muscles/group. *Significantly different from control (P < 0.05).

Soleus muscles isolated from weight bearing and cast immobilized rats were weighed to confirm muscle atrophy, and immediately placed in liquid nitrogen. Skeletal muscle atrophy increased progressively in response to hind limb cast immobilization. Indeed, soleus muscle mass was decreased by 13%, 26%, and 37% in response to 2, 4 and 6 days of cast immobilization, respectively (**Fig 2A**). We also harvested the plantaris muscle and measured the mRNA level of two commonly used biomarkers of skeletal muscle atrophy, MAFbx/atrogin-1 and MuRF1, which are increased in virtually all models of muscle atrophy and are required for normal muscle atrophy, at least during denervation [18, 39, 40]. Atrogin-1 was increased 3.5, 4.0 and 2.0 fold and MuRF1 increased by 3.7, 4.1 and 1.6 fold at 2, 4 and 6 days of cast immobilization, respectively (**Fig 2B**). These results validate the findings of the acetylome and ubiquitinome analysis, presented below, by confirming normal activation of the atrophy program in the hind limb muscles.

### Acetylome

#### Global view

We identified a total of 425 proteins which were acetylated in skeletal muscle during normal, weight-bearing conditions. Within these 425 proteins we identified 1,326 lysine acetylation sites (**S1 and S2 Tables**). To identify the computationally derived biological processes of these acetylated proteins we functionally classified them using the DAVID Bioinformatics database. The top 5 annotated clusters demonstrate that a large proportion of acetylated proteins are proteins of the mitochondrial inner membrane, the contractile fiber and metabolic processes (**Table 1**). The high abundance of acetylated mitochondrial proteins is consistent with previously published acetylome data in liver [41] and recently published acetylome data in rat and human skeletal muscle during normal physiological conditions [42] in which many mitochondrial enzymes and proteins are regulated via acetylation/deacetylation [42–45]. The large number of acetylated contractile proteins is also consistent with recently published work in skeletal muscle [42]. Indeed more than 80% of contractile proteins are acetylated in skeletal muscle during normal physiological conditions, a pattern that is common between rat and human skeletal muscle [42].

**Table 1:** Gene Ontology functional annotation clusters of acetylated proteins in skeletal muscle during normal weight-bearing conditions.

Functional Annotation Cluster	Number of Acetylated Proteins
Mitochondrial Membrane	72
Contractile Fiber	32
TCA Cycle	19
ATP Metabolic Processes	23
Glycolysis	18

Of the 425 acetylated proteins, 43, 47 and 50 were differentially acetylated (increased or decreased), based on the criteria specified in the methods, at 2, 4 and 6 days of immobilization, respectively, compared to weight bearing controls (**S3 Table**). Of these differentially acetylated proteins multiple acetylation sites (>1) were identified in 14 proteins. Among these 14 proteins only histone H2B and myosin 2 (myosin heavy chain IIa) /myosin 7 (myosin heavy chain I) (the peptide sequence homology does not allow us to distinguish between the two) contained more than 5 differentially acetylated sites. Interestingly, at each time point the majority of the differentially acetylated proteins showed a *decrease* in acetylation, particularly at 2 and 6 days of immobilization, suggesting that during disuse atrophy acetylated skeletal muscle proteins become predominantly deacetylated. However we also observed several hyper-acetylated peptides, demonstrating that acetylation-deacetylation is not a global cellular event, but rather a targeted specificity by HAT and HDAC enzymes.

#### Gene ontology analysis of differentially acetylated proteins

**Fig 3:**
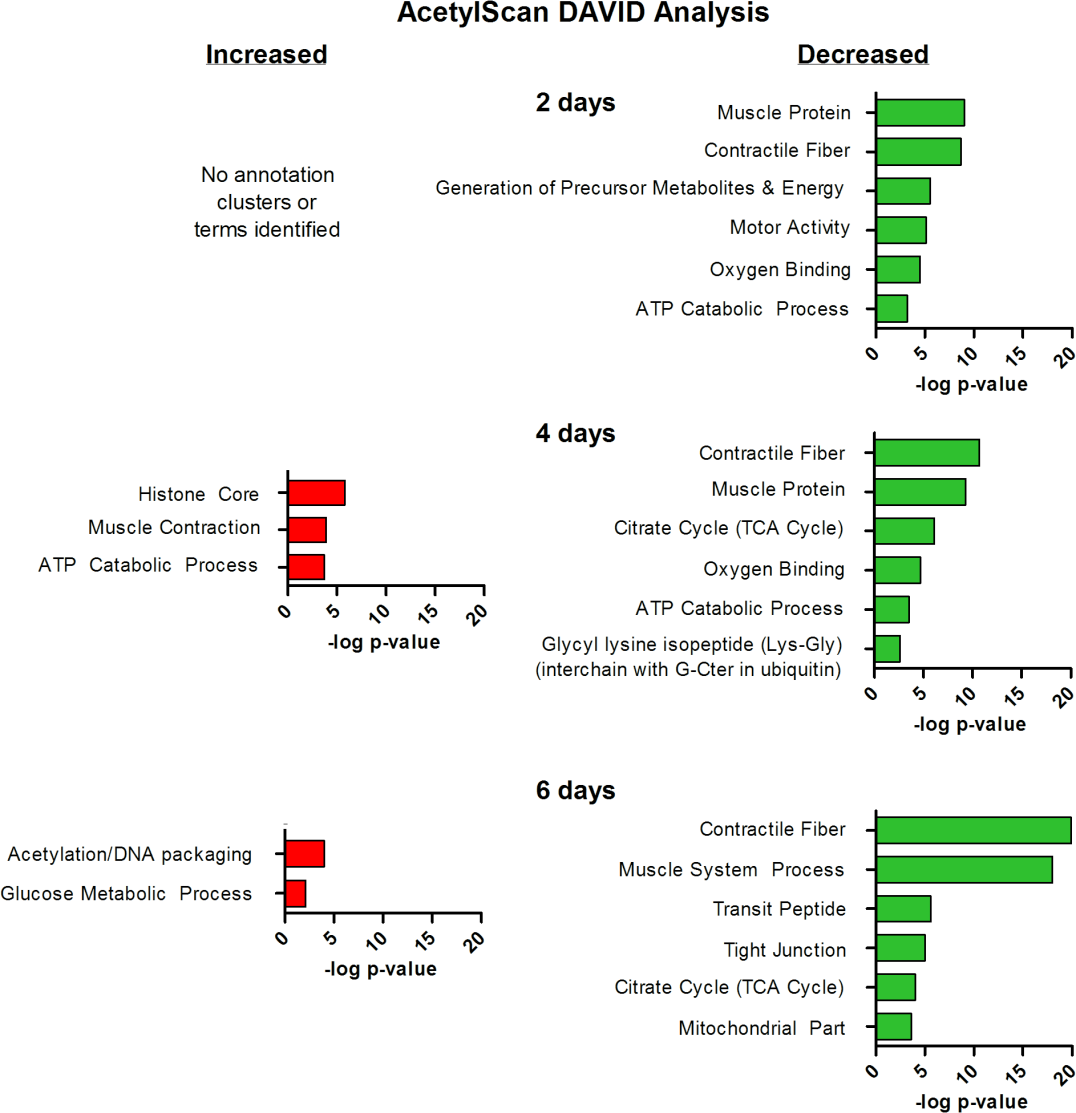
Pathway analysis of muscle proteins differentially acetylated following 2-, 4- and 6-days of disuse. Muscle proteins containing sites of increased or decreased acetylation in response to muscle disuse were analyzed using the DAVID functional annotation clustering module to identify enriched biological processes. Clusters are ranked in order of significance and are plotted against the-log of the p-value.

In order to identify the computationaly derived biological pathways enriched by differentially acetylated proteins during muscle disuse the proteins increased and decreased in acetylation at each time point were functionally categorized using the DAVID Bioinformatics database. Enriched annotation clusters for proteins showing increased or decreased acetylation at 2, 4 and 6 days are shown in **Fig 3**, and the comprehensive list of significantly enriched clustered annotations, and the proteins annotating to these terms are shown in **S4 Table.** Since there were only 7 proteins which showed an increase in acetylation at 2 days of disuse no enriched annotation terms or clusters were identified. As shown in **Fig 3**, for proteins increased in acetylation at 4 and 6 days of muscle disuse the most highly enriched annotation clusters were related to *histone core* and *acetylation/DNA packaging*. Among the proteins annotating to these functional clusters are several related to the DNA binding protein class including histone H2B, Histone H2A.Z (which is one of the 5 major variants of histone H2A), histone H3, and histone H4. Although the physiological significance of the increased acetylation of these proteins in the current study cannot be inferred, acetylation of histones is known to neutralize their positive charge, weakening the electrostatic attraction between histone and negatively charged DNA, thereby loosening the chromatin structure to generally increase gene transcription [46–48]. This increase in histone acetylation during muscle disuse could, therefore, allow for increased transcription of genes involved in the muscle atrophy process.

For proteins showing a decrease in acetylation the most highly enriched annotation clusters were related to muscle contraction and energy metabolism (**Fig 3**). Proteins annotated to clusters related to muscle contraction include alpha actin, alpha actinin, myosin light chains 1 (MLC1) and 3 (MLC3), troponin C, troponin T, and several myosin heavy chain isoforms, including myosin 7 (myosin heavy chain I) and myosin 2 (myosin heavy chain IIa), which are the predominant myosin heavy chain isoforms in the rat soleus muscle. Three lysine residues within myosin 2 and/or myosin 7 showing decreased acetylation lie within the motor domain (amino acids 1–786), which is consistent with data from cardiomyocytes in which the motor domains of both alpha-myosin and beta-myosin (fast and slow cardiac isoforms) are modified via lysine acetylation [49]. The single acetylation site in MLC1 and both acetylation sites in MLC3 showing decreased acetylation during disuse are within the N terminus, which binds to the C terminus of actin and may play an important modulatory role in actin-activated myosin adenosine triphosphatase (ATPase) activity, shortening velocity and power output [50–52]. Therefore decreased acetylation within this region could regulate these functions. Troponin C type 1 (Tnnc1) protein acetylation also decreased, following 2 and 6 days of disuse. Troponin C is a calcium binding protein central to the regulation of skeletal muscle contraction and its acetylation status has previously been shown to increase its stability and interactions with calcium [53].

Proteins showing decreased acetylation which annotate to clusters related to energy metabolism include several TCA cycle enzymes including aconitate hydratase, isocitrate dehydrogenase 2-oxyglutarate dehydrogenase and succinate dehydrogenase, as well as ATP synthase subunit beta (ATP5B) and O (ATP5O) which are subunits of oxidative phosphorylation complex V. Given that these enzymes are localized to the mitochondria their deacetylation is potentially mediated via Sirtuin3 (SIRT3) which is a major mitochondrial deacetylase [54]. Indeed SIRT3 has been shown to deacetylate and activate several mitochondrial proteins including isocitrate dehydrogenase [55, 56], succinate dehydrogenase [57] and physically interact with ATP5O [57].

### Ubiquitinome

#### Global view

Despite the long standing knowledge that ubiquitination is a widespread protein modification which regulates diverse biological pathways and is critical to protein degradation during muscle atrophy, a comprehensive understanding of ubiquitinated proteins and their modification sites in skeletal muscle during any physiological or pathophysiological condition is currently lacking. This is likely due, at least in part, to methodological limitations due to the low occupancy of ubiquitination on endogenous proteins. This limitation has been circumnavigated by many via overexpression of exogenous epitope-tagged ubiquitin [58]; though, the extent to which this affects the occupancy and specificity of ubiquitination is not clear. In the current study we take advantage of recent advances in enrichment strategies by utilizing an antibody that recognizes endogenous ubiquitin branch (K-ε-GG)-containing peptides. These di-Gly remnants are derived from the trypsinolysis of ubiquitin conjugates due to the cleavage of ubiquitin’s C-terminal Arg-Gly-Gly sequence which leaves Gly-Gly still covalently attached to the target lysine residue via an isopeptide bond [59]. This signature peptide causes a mass shift at the lysine residue which we subsequently quantified via LC-MS/MS and then we identified the peptide using the database searches outlined in the methods. It is important to acknowledge that, in addition to ubiquitin, NEDD8 and ISG15, which are ubiquitin-like proteins (UBLs), also contain C-terminal di-Gly motifs which are generated by trypsin cleavage. Therefore assessment of the di-Gly modified proteome is a composite of proteins modified by ubiquitin as well as NEDD8 and ISG15. However, in a recent study using the same methodology as used in the current work, >94% of K-ε-GG sites were shown to be the result of ubiquitination as opposed to Nedd8ylation or ISG15ylation [59, 60]. It is therefore likely that the vast majority of the di-Gly modified peptides identified in the current study also reflect ubiquitin modification. Therefore for simplicity, and similar to others, we refer to the di-Gly modified proteins in the current as ubiquitin-modified proteins.

Using this strategy the ubiquitinome screen identified 1,131 ubiquitin-modified proteins in skeletal muscle during normal, weight-bearing conditions, containing 4,948 lysine ubiquitination sites (**S5 and S6 Tables**). To identify the computationally derived biological processes enriched by ubiquitinated proteins during normal control conditions we functionally classified the 1,131 proteins using the DAVID Bioinformatics database, similar to our analysis of the acetylome data. The top 5 annotated clusters are shown in **Table 2**, with *contractile fiber* identified as the most enriched cluster of ubiquitinated proteins, followed by *proteolysis involved in cellular protein catabolic processes*. Annotated to this latter category were multiple proteasomal subunits, ubiquitin conjugating enzymes, ubiquitin E3 ligases, and ubiquitin shuttling proteins. This is consistent with data obtained in HEK293 cells using comparable methods which identified the ubiquitination of many ubiquitin-conjugating enzymes, ubiquitin ligases and 26S proteasome regulatory subunits [61]. Moreover, several components of the human 26S proteasome were recently shown to be ubiquitinated including the ubiquitin receptors Adrm1 and S5a/Psmd4, the ATPase subunit Rpt5 and the deubiquitinating enzyme Uch37/UchL5, which is important in the disassembly of the ubiquitin chain necessary for subsequent substrate entry into the gated channel of 20S proteasome [62, 63]. Functionally, the ubiquitination of Adrm1, S5a/Psmd4, Rpt5 and Uch37/UchL5 was associated with an impaired ability of the 26S proteasome to bind, deubiquitinate and degrade ubiquitinated proteins [62]. In the current study each of these proteins was ubiquitinated under control conditions on at least one lysine residue, with S5/Psmd4 ubiqutinated on 8 residues and Rpt5 on 6 residues. It is therefore possible that these modifications serve to reduce activity of the 26S proteasome during basal conditions.

**Table 2:** Gene Ontology functional annotation clusters of ubiquitinated proteins in skeletal muscle during normal weight-bearing conditions.

Functional Annotation Cluster	Number of Acetylated Proteins
Contractile Fiber	51
Proteolysis involved in cellular protein catabolic process	39
Heart Contraction	11
Muscle Organ Development	26
Cellular Carbohydrate Catabolic Process	16

Of the 1,131 total ubiquitin-modified proteins, 183, 222 and 172 were differentially ubiquitinated (increased or decreased) based on the criteria specified in the methods, at 2, 4 and 6 days of immobilization, respectively, compared to weight bearing controls (**S7 Table**). Multiple ubiquitination sites (≥2) were found in 87 proteins, of which 26 proteins contained >5 ubiquitination sites, 21 of which were sarcomeric proteins important to structure and/or contraction. Interestingly, of the differentially ubiquitinated proteins, more proteins showed a *decrease* in ubiquitination at each time point than showed an *increase*. However, proteins showing an increase in ubiquitination contained substantially more ubiquitination sites and thus the total number of sites showing an increase in ubiquitination was substantially greater (613 sites) than the number of sites showing a decrease in ubiquitination (306). It is also notable that not all ubiquitination events on protein substrates are regulated similarly. For example on nebulin, 15 lysine sites showed an increase, and 7 lysine sites a decrease, in ubiquitination in response to disuse. Such differential regulation at different sites could make it difficult to detect changes in nebulin ubiquitination using the more traditional immunoprecipitation and subsequent western blotting approach which measures only global changes in protein ubiquitination.

#### Gene ontology analysis of differentially ubiquitinated proteins

**Fig 4:**
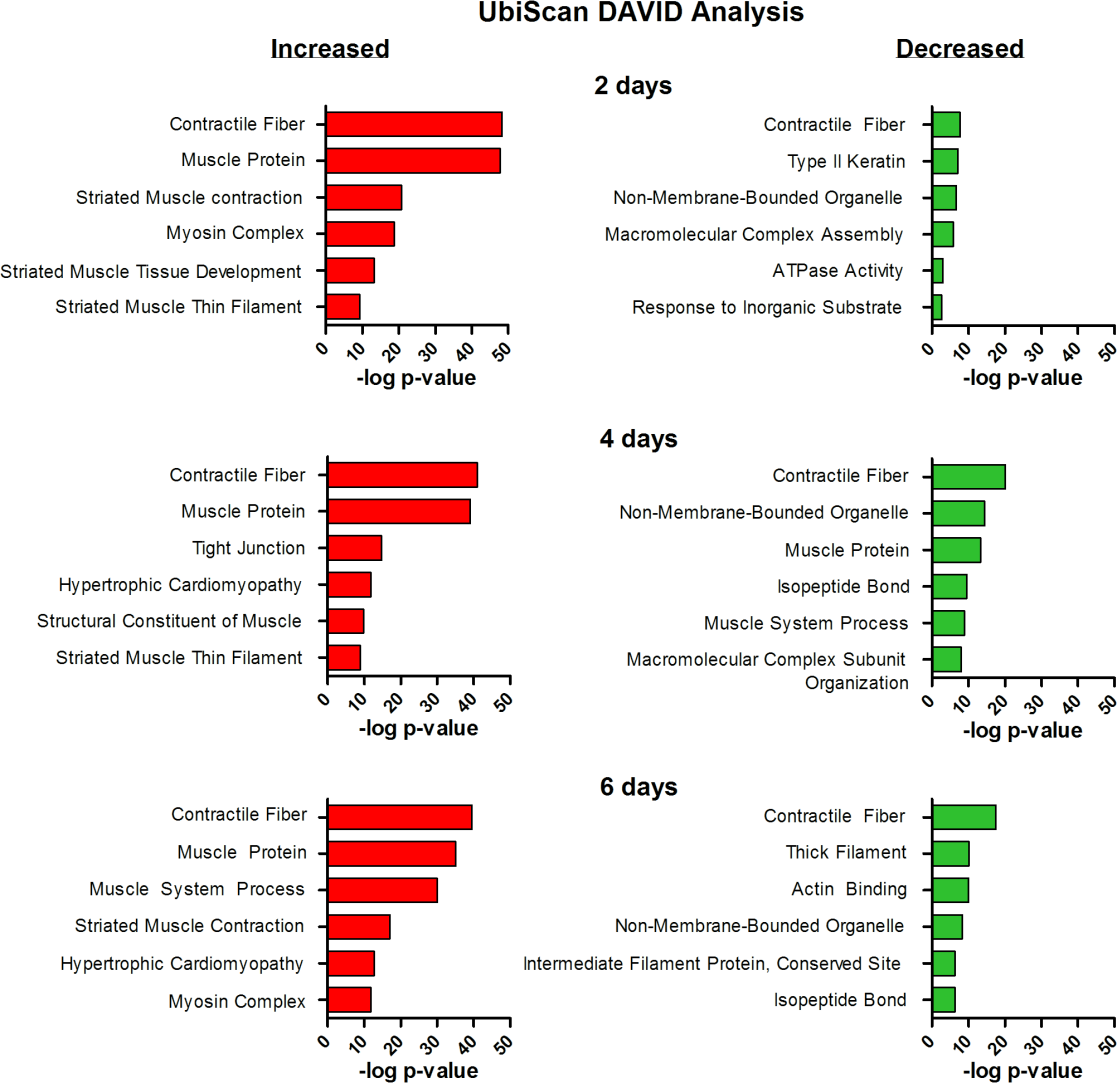
Pathway analysis of muscle proteins differentially ubiquitinated following 2-, 4- and 6-days of disuse. Muscle proteins containing sites of increased or decreased ubiquitination in response to muscle disuse were analyzed using the DAVID functional annotation clustering module to identify enriched biological processes. Clusters are ranked in order of significance and are plotted against the-log of the p-value.

The enriched annotation clusters for proteins increased and decreased in lysine ubiquination are shown in **Fig 4** and show that *contractile fiber* is the most highly enriched category at all time-points for proteins increased and decreased in ubiquitination. However the enrichment for *contractile fiber* is substantially greater for proteins increased in ubiquitination compared to proteins decreased in ubiquitination. Within the contractile fiber category a vast number of the proteins increased in ubiquitination are sarcomeric, including alpha actin, myosin-7 (the isoform found in type I fibers), myosins 1, 2, 4 and 8 (the isoforms found in type II fibers), myosin light chain 3, tropomyosin, troponins C, I and T, and titin (**S8 Table**). In fact, of all proteins, titin showed the greatest number of ubiquitinated lysine residues with 186, of which 173 showed increased ubiquitination at least at one time point compared to control. Titin is the largest known protein consisting of 34,350 amino acids and the third most abundant muscle protein after myosin and actin [64]. In addition to being a critical sarcomeric structural protein, with its N-terminal integrated into the Z-line region and the C-terminal connected to the M-line region, titin also plays a critical role in the passive stiffness of muscle [65] and in signal transduction [66]. Although titin is known to undergo phosphorylation [65, 67–70] and recently MuRF1-dependent ubiquitination [71], to our knowledge this is the first evidence demonstrating its differential ubiquitination in response to any physiological perturbation as well as the first data to identify the specific ubiquitination sites. Interestingly only 3 lysine sites in the Z-disk region of titin and 6 in the I-band region showed an increase in ubiquitination, whereas the A-band and M-line regions contained 118 and 46 lysine sites which showed an increase in ubiquitination, respectively (**S7 Table**). Given that M-line titin only includes ~2,500 amino acids compared to A-band titin’s ~18,000 amino acids, the M-line seems to show particular enrichment for ubiquitination. In support of this another M-line protein, myomesin-1, which contains 1,451 amino acids and binds the C-terminal end of titin, also showed increased ubiquitination at 24 unique lysine residues.

The increased ubiquitination of myosin in response to muscle disuse was similarly extensive. Indeed, within myosin 7 there were 33 lysine sites which showed an increase in ubiquitination and an additional 45 lysine sites identified in peptides which mapped to myosin 7 (as well as other myosin isoforms due to the sequence homology). Interestingly, 6 lysine sites within the motor domain of myosin 7 showed increased ubiquitination and 5 sites showed decreased ubiquitination. These findings demonstrate that the motor domain of myosin 7 is regulated via ubiquitination, just as it is regulated via acetylation as determined in the acetylome analysis.

As mentioned above, the most highly enriched annotation cluster for proteins showing a decrease in ubiquitination was also *contractile fiber*. However, there were substantially fewer proteins and lysine sites which annotated to *contractile fiber* among the proteins that were decreased in ubiquitination compared to those that were increased in ubiquitination. For example, within this category after 2 days of disuse, 32 lysine sites in 18 proteins showed decreased ubiquitination whereas 305 lysine sites in 32 proteins showed increased ubiquitination. Included among the proteins showing decreased ubiquitination of at least one or more lysine residues were alpha actin, myosin 7 and myosin 2, titin, and troponin I (**S7 Table**). However, since the majority of these proteins were identified to show increased ubiquitination at other lysine residues, these data strongly support protein ubiquitination as a highly specific process that may have lysine-specific effects.

Another enriched annotation cluster at 2, 4 and 6 days of disuse for proteins showing a decrease in ubiquitination is *non-membrane-bounded organelle*. Annotated to this category include Histone H2A and Histone H2B, both of which are known to be predominantly monoubiquitinated, although they can also be modified by polyubiquitin chains [72, 73]. The ubiquitination of histones is known to regulate several cellular processes including transcription, maintenance of chromatin structure and DNA repair [73]. It is therefore possible that the decreased ubiquitination of H2A and H2B during muscle disuse is a mechanism to regulate gene transcription, perhaps of atrophy-related genes. Alternatively, since there is a limited pool of free ubiquitin, deubiquitination of histones is suggested to be a rapid mechanism to recycle ubiquitin for subsequent polyubiquitination and degradation of proteins by the proteasome during times of cellular stress [72]. Under this scenario increased UPP-mediated protein degradation may be coupled to deubiquitination of histones, chromatin remodeling and significant changes in gene transcription, as has been shown by others [74, 75].

### Crosstalk between Acetylation and Ubiquitination

Since lysine residues can be modified by both acetylation and ubiquitination we determined the extent to which the lysine residues and proteins identified to be modified in our study were modified by both posttranslational modifications during the progression of disuse muscle atrophy. As shown in **S9 Table**, 23 proteins were differentially modified by both acetylation and ubiquitination during muscle disuse. However, within these proteins only 7 lysine sites showed a coordinate decrease in acetylation and an increase in ubiquitination while just 1 site showed an increase in acetylation and a decrease in ubiquitination **Table 3** Therefore, while direct competition between acetylation and ubiquitination of these specific lysine residues may occur during the disuse period, given the total number of lysine sites which were regulated through acetylation or ubiquitination, this direct competition does not seem to be extensive. However, the interplay between post translational modifications goes beyond direct competition. Indeed the “posttranslational code” suggests that posttranslational modifications are codified and occur in sequential combinations [76–78]. It is therefore possible that this code is in effect for all 23 proteins differentially acetylated and ubiquitinated.

**Table 3:** Lysine sites identified as differentially acetylated and ubiquitinated (bolded numbers) during muscle disuse for which direction of change was different for each modification.

		AcetylScan			UbiScan		
Protein	Lysine Site	2 days	4 days	6 days	2 days	4 days	6 days
Actinin alpha 2	432	-1.3	-1.9	**-3.4**	**3.8**	**3.5**	**3.5**
Hemoglobin subunit beta-2	45	1.0	-1.1	**2.1**	-1.9	**-4.5**	-1.8
Myosin 2	786	**-2.0**	**-2.0**	**-2.9**	**2.5**	1.3	1.0
	1463	**-3.0**	**-3.4**		**2.9**	**3.4**	1.7
	1776	-1.6	-1.6	**-2.3**	1.8	**3.0**	1.3
Myosin 7	1225	**-2.3**	-1.7	-1.3	**2.1**	**3.8**	1.5
	1770	-1.6	-1.6	**-2.3**	1.8	**3.0**	1.3
Troponin T, slow	166	**-2.1**	-1.9	-1.7	1.7	**3.0**	**2.5**

**Fig 5:**
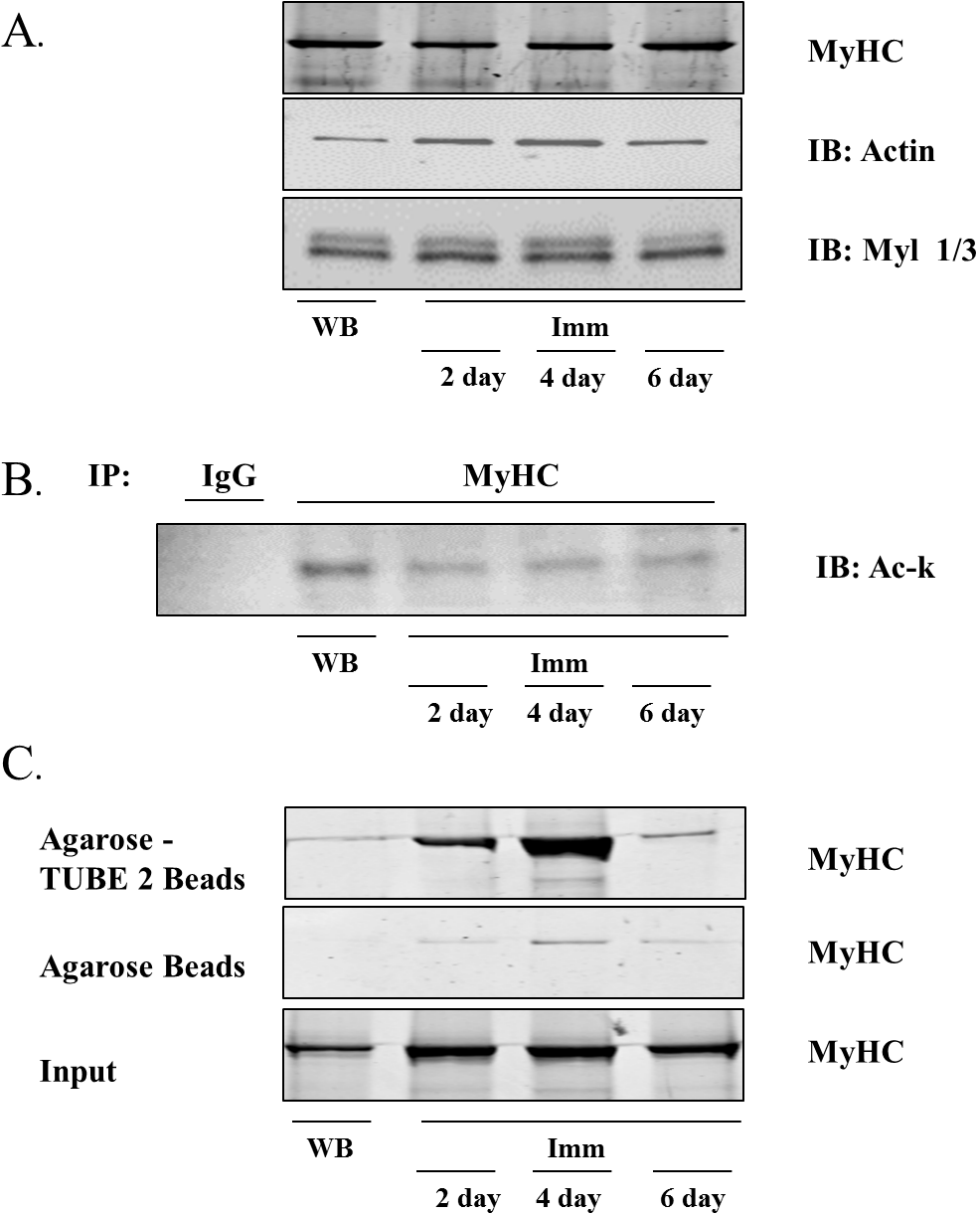
Myosin Heavy Chain (MyHC) levels, acetylation and ubiquitination in response to muscle disuse. **A)** Representative blots of MyHC, actin and myosin light chain (Myl) 1/3 protein expression in weight bearing (WB) control and 2, 4 and 6 day cast immobilized (Imm) muscles. **B)** The acetylation of MyHC was determined by incubating equal amounts of protein extract with an antibody against MyHC to immunoprecipitate (IP) MyHC or IgG as a negative control. Precipitated proteins were subjected to SDS-PAGE and immunoblotted for acetylated protein using an antibody against acetyl-lysine (Ac-k). **C)** Immunoprecipitation based enrichment of ubiquitinated proteins, pulled down by Agarose-TUBE2 beads. Precipitates were run on a polyacrylamide gel and the gel stained with Coomassie blue to visualize MyHC. Experiments in B and C were independently repeated three times.

The predominant decrease in protein acetylation during disuse could be interpreted to be due to a preferential decrease in the expression level of these proteins. We therefore measured the expression of three proteins which showed preferential deacetylation during muscle disuse—myosin heavy chain (MyHC), actin and myosin light chain 1/3. As shown in **Fig 5A** when loading equal amounts of protein, the relative levels of each protein remained unchanged at each time point, verifying that the decreased acetylation of these proteins, at these time points, is not simply due to a preferential decrease in their levels. Moreover, there are multiple further lines of evidence which refute, on a broader scale, this possibility that a preferential decrease in protein level might explain the predominant decrease in acetylation. First, several proteins show decreased acetylation at one lysine residue and increased acetylation at another lysine (e.g. myosin 2, serum albumin precursor). Second, the magnitude of change differs across time within the same protein (e.g. carbonic anhydrase K212: 1.3-fold, -4.1-fold and -1.2-fold after 2, 4 and 6 days of disuse). Third, several proteins show different fold changes at different lysine residues within the same protein (e.g. 2-oxogluterate dehydrogenase, K276: -8.8, -8.0 and -5.0; K582, -1.7, -4.2, -2.8, following 2, 4 and 6 days of disuse, respectively. Fourth, several deacetylated proteins showed increased ubiquitination (e.g. alpha actin: decreased acetylation of K70 and K286, increased ubiquitination of K50, K61, K191, K326, K328 and K336). Thus, when all the data are considered together, it is clear that while changes in specific protein levels should be considered when interpreting the data, changes in protein level alone cannot account for the changes in acetylation and ubiquitination.

Since MyHC showed extensive deacetylation and increased ubiquitination in the acetylome and ubiquitinome studies, respectively, we determined if these modifications could also be detected using traditional co-immunoprecipitation procedures. Indeed, as shown in **Fig 5B**, MyHC is acetylated during normal weight bearing (control) conditions and its acetylation is decreased in response to 2, 4 and 6 days of muscle disuse. Further, as shown in **Fig 5C**, MyHC is also ubiquitinated during normal weight bearing conditions and its ubiquitination is substantially increased in response to muscle disuse, particularly after 2 and 4 days.

## Discussion

Acetylation plays a major role in regulating protein stability, localization and function [79], and is therefore a key mechanism regulating cell biology. Moreover, in recent years the modification of proteins through acetylation has emerged as a key mechanism regulating skeletal muscle mass during various pathophysiological conditions [9–12, 16]. However, despite the critical roles of protein acetylation in the regulation of cellular homeostasis during control and pathophysiological conditions, our understanding of the specific proteins modified via reversible lysine acetylation in skeletal muscle, just as in other tissues, is in its infancy. Indeed, the first comprehensive analysis of protein acetylation in skeletal muscle during normal physiological conditions was only recently published in 2012 by Lundby et al [42]. However, the skeletal muscle proteins exhibiting changes in lysine acetylation in response to any physiological or pathophysiological condition are largely unknown, with the exception of a small number of proteins identified in isolated studies. In order to better understand the role of protein acetylation as a mechanism to control skeletal muscle biology, the proteins modified via changes in lysine acetylation during these conditions must first be identified. In the current study we therefore provide the first comprehensive proteomic analysis of skeletal muscle proteins modified via changes in lysine acetylation in response to any physiological or pathophysiological condition. Specifically, we identified the skeletal muscle proteins modified via reversible acetylation during the progression of skeletal muscle atrophy induced by muscle disuse. In control muscle tissue, we identified a total of 452 acetylated proteins containing 1,318 acetylation sites, indicating that lysine acetylation may be involved in regulating the basal function of these proteins, and thus the regulation of normal cellular homeostasis in skeletal muscle. Of these 452 proteins, 49, 52 and 55 were differentially acetylated following 2, 4 and 6 days of immobilization, respectively, which suggests that the acetylation of these proteins may play an important role in regulating cellular processes consequent to muscle disuse, including atrophy, weakness, and metabolic shifts [23, 80, 81].

During control conditions a large number of proteins annotating to the contractile fiber were acetylated, which is in close agreement with recently published work in rats and humans which showed that more than 80% of proteins involved in striated muscle contraction are acetylated [42]. Furthermore, biological processes related to striated muscle contraction were one of the largest categories identified through gene ontology to be differentially acetylated during muscle disuse, with a predominant decrease in acetylation at 2, 4 and 6 days of disuse. Although, to our knowledge, there are no data on the physiological relevance of contractile protein acetylation in skeletal muscle, acetylation of cardiac contractile proteins has been shown to positively regulate cardiac contractile function [82]. Indeed, in cardiomyocytes the acetyltransferase, p300/CBP-associated factor (PCAF) and the deacetylases HDAC3 and HDAC4 associate with cardiac sarcomeres, and acetylate and deacetylate myosin heavy chain (MHC), respectively [49, 83]. Importantly, this reversible acetylation regulates myosin ATPase activity and thin filament sliding velocity, with acetylation enhancing both of these variables [49]. Moreover, increasing MHC acetylation, through inhibition of HDACs or treatment with acetyl-CoA [49], increases calcium sensitivity of cardiac myofilaments [83]. Together, these data support a role for MHC acetylation in enhancing contractile performance in cardiomyocytes. Therefore our finding that skeletal muscle contractile proteins are deacetylated during disuse suggests this may contribute to the associated contractile dysfunction [81]. In support of this, recent work from our lab demonstrates that treatment of cast immobilized mice with MS-275 (Entinostat), a class I HDAC inhibitor, completely prevents the decrease in specific force caused by 10 days of disuse [10]. Moreover, MS-275 treatment also prevented the preferential decrease in myosin heavy chain protein expression that occurs at later time points of skeletal muscle disuse. Based on these combined findings, it may be speculated that deacetylation of myosin heavy chain increases its susceptibility to ubiquitination and degradation, thus contributing to muscle weakness.

In addition to myosin heavy chain, several other contractile proteins were also differentially acetylated and ubiquitinated during disuse. This enrichment of contractile proteins may be a function, in part, of the immunoprecipitation and mass spectrometry methodology. Indeed both methods are concentration sensitive and, therefore, well suited to detect contractile proteins, which comprise ~50–60% of total muscle proteins. By the same token the methodology is less well suited to the detection of lower abundant proteins, such as transcription factors. Therefore while the number of acetylated and ubiquitinated proteins identified in the current study is vast, it likely represents only a subset of the total cellular proteins modified. Therefore subsequent proteomic analyses of acetylated and ubiquitinated peptides from cytosolic and nuclear fractions in which myofibrillar proteins have been removed would perhaps reveal an additional dataset to include transcription factors and other signaling molecules whose expression levels are much lower. Moreover, the experimental design of the ubiquitinome assessment means that proteins which are rapidly degraded after ubiquitination would not be detected. However the detection of such proteins would require their stabilization by chemical proteasome inhibition, which in the current study could have interfered with lysine acetylation.

Another restriction of the methodology used for the ubiquitinome assessment in the current study is that it does not distinguish between lysine residues which have been modified by a single ubiquitin molecule (monoubiquitinated) and those which have been modified by a polyubiquitin chain. However this is also a restriction when determining protein ubiquitination using less sensitive methods such as western blot or immunoprecipitation combined with western blot. Functionally, knowledge of monoubiquitination versus polyubiquitination is important because monoubiquitination regulates receptor transport, viral budding and DNA repair [84–87], whereas 26S proteasome-mediated protein degradation is regulated predominantly by Lys-48 polyubiquitin chains. In addition to K48, ubiquitin can also conjugate to six other lysine residues (K6, K11, K27, K29 and K63) to form polyubiquitin chains that are homogenous, mixed or branched, and in the current study we detected ubiquitination of all seven lysine residues on ubiquitin, during both control and disuse conditions. In fact, the signal intensity from K48-Ub was the highest of all peptides during control conditions (>439,000,000) and the second highest of all peptides at 2, 4 and 6 days of disuse. Although we did not detect any changes in K48-Ub during muscle disuse, this is likely due to the proteasomal degradation of K48-Ub modified proteins during the progression of muscle disuse and due to the high signal intensity during control conditions, thus making it difficult to detect an increase. In fact we did not find changes in the ubiquitination of ubiquitin at any of its lysine residues which, again, may be related to the very high signal intensity at each lysine during control conditions (K6, >14,000,000; K11, >49,000,000; K27, >6,900 000; K29 >2,400,000; K63, >117,000,000).

## Conclusions

The data in the current study provide the first comprehensive analysis of acetyl-modified and ubiquitin-like modified proteome, within the same samples during a physiological condition of muscle wasting. The major implications of our finding are twofold. First, since a multitude of contractile proteins are both deacetylated and ubiquitinated during the progression of muscle disuse, we speculate that deacetylation of contractile proteins is linked to their subsequent ubiquitination and degradation. This speculation is based on the knowledge that a) myofibrillar proteins make up over a half of total muscle proteins and decrease to a greater extent than soluble proteins during disuse atrophy conditions [88], b) the ubiquitin proteasome pathway is the predominant pathway of protein turnover during atrophy conditions [18], and c) inhibition of various HDACs attenuates disuse muscle atrophy and the loss of myosin heavy chain [9, 10, 12]. While this may be mediated by direct competition in that a deacetylated lysine is free to become ubiquitinated, our data suggest a more likely scenario is via the posttranslational code in which one post translational modification may encode for another post-translational modification event at adjacent or distal residues. The second major finding from the current study is of decreased ubiquitination and increased acetylation of histones. Such covalent modifications of histone proteins are well known to play critical roles in the regulation of chromatin remodeling and gene expression via three main mechanisms. The first is via alterations to the electrostatic charge of histones, which may alter chromatin structure and its interaction with DNA thereby altering access of the transcriptional machinery to gene promoters [46–48]. The second is via the recruitment of regulatory proteins that affect gene transcription [89, 90], and the third is based on the histone code hypothesis. This code suggests that covalent modification of histone tails, including acetylation and ubiquitination, has much broader implications and that epigenetic modifications occur in combinations to considerably extend the genetic code to potentially impact all chromatin-templated processes [91–95]. While more work is clearly necessary to explore these possibilities, the current data provide a substantial resource for subsequent mechanistic studies which determine the physiological significance of acetylation and ubiquitination of specific skeletal muscle proteins in the regulation of the muscle atrophy phenotype.

## Supporting Information

S1 Table All identified acetylation sites. (XLSX)

S2 Table Spectra of all identified acetylation sites. (XLSX)

S3 Table Sites differentially acetylated in response to 2-, 4 and/or 6-days of cast immobilization. (XLSX)

S4 Table Differentially acetylated proteins annotating to the enriched Gene Ontology clusters presented in Fig 3. (XLSX)

S5 Table All identified ubiquitin-like modified sites. (XLSX)

S6 Table Spectra of all identified ubiquitin-like modified sites. (XLSX)

S7 Table Sites differentially ubiquitinated in response to 2-, 4 and/or 6-days of cast immobilization. (XLSX)

S8 Table Differentially ubiquitinated proteins annotating to the enriched Gene Ontology clusters presented in Fig 4. (XLSX)

S9 Table Proteins differentially acetylated and ubiquitinated in response to 2-, 4 and/or 6-days of cast immobilization. (XLSX)
